# Digital Education for the Management of Chronic Wounds in Health Care Professionals: Protocol for a Systematic Review by the Digital Health Education Collaboration

**DOI:** 10.2196/12488

**Published:** 2019-03-25

**Authors:** Laura Martinengo, Natalie Jia Ying Yeo, Zheng Qiang Tang, Kasturi D/O Markandran, Bhone Myint Kyaw, Lorainne Tudor Car

**Affiliations:** 1 Centre for Population Health Sciences Lee Kong Chian School of Medicine Nanyang Technological University Singapore Singapore; 2 Laboratory of Molecular and Vascular Medicine Lee Kong Chian School of Medicine Nanyang Technological University Singapore Singapore; 3 Dermatology and Skin Biology Lee Kong Chian School of Medicine Nanyang Technological University Singapore Singapore; 4 Metabolic Disorders Lee Kong Chian School of Medicine Nanyang Technological University Singapore Singapore; 5 Family Medicine and Primary Care Lee Kong Chian School of Medicine Nanyang Technological University Singapore Singapore; 6 Department of Primary Care and Public Health School of Public Health Imperial College London London United Kingdom

**Keywords:** distance education, digital education, e-learning, continuous medical education, health professions, health personnel, leg ulcers, pressure ulcers, systematic review

## Abstract

**Background:**

Digital education is “the act of teaching and learning by means of digital technologies.” Digital education comprises a wide range of interventions that can be broadly divided into offline digital education, online digital education, digital game-based learning, massive open online courses (MOOCs), psychomotor skills trainers, virtual reality environments, virtual patient simulations, and m-learning. Chronic wounds pose an immense economic and psychosocial burden to patients and the health care system, as caring for them require highly specialized personnel. Current training strategies face significant barriers, such as lack of time due to work commitments, distance from provider centers, and costs. Therefore, there is an increased need to synthesize evidence on the effectiveness of digital education interventions on chronic wounds management in health care professionals.

**Objective:**

Our main objective is to assess the effectiveness of digital education as a stand-alone approach or as part of a blended-learning approach in improving pre- and postregistration health care professionals’ knowledge, attitudes, practical skills, and behavior in the management of chronic wounds, as well as their satisfaction with the intervention. Secondary objectives are to evaluate patient-related outcomes, cost-effectiveness of the interventions, and any unfavorable or undesirable outcomes that may arise.

**Methods:**

This systematic review will follow the methodology as described in the Cochrane Handbook for Systematic Reviews of Interventions. As our systematic review is one of a series of reviews on digital education for health professionals’ education, we will use a previously developed search strategy. This search includes the following databases: the Cochrane Central Register of Controlled Trials (CENTRAL) (Cochrane Library), MEDLINE (Ovid), Embase (Ovid), Web of Science, the Educational Resource Information Centre (ERIC) (Ovid), PsycINFO (Ovid), the Cumulative Index to Nursing and Allied Health Literature (CINAHL) (EBSCO), the ProQuest Dissertation and Theses database, and trial registries. Databases will be searched for studies published from January 1990 to August 2018. Two independent reviewers will screen the library for included studies. We will describe the screening process using a flowchart as per the Preferred Reporting Items for Systematic Reviews and Meta-Analyses (PRISMA) guidelines. We will extract the data using a previously developed, structured data extraction form. Included studies will be quality-assessed using the Risk of Bias tool from Cochrane. We will narratively summarize the data and, if possible, we will conduct a meta-analysis. We will use Cochrane’s RevMan 5.3 software for data analysis.

**Results:**

We have completed the screening of titles and abstracts for this systematic review and are currently selecting papers against our inclusion and exclusion criteria through full-text revision. We are expecting to complete our review by the end of April 2019.

**Conclusions:**

This systematic review will provide an in-depth analysis of digital education strategies to train health care providers in the management of chronic wounds. We consider this topic particularly relevant given the current challenges facing health care systems worldwide, including shortages of skilled personnel and a steep increase in the population of older adults as a result of a prolonged life expectancy.

**Trial Registration:**

PROSPERO CRD42018109971; https://www.crd.york.ac.uk/prospero/display_record.php?RecordID=109971

**International Registered Report Identifier (IRRID):**

DERR1-10.2196/12488

## Introduction

Since the beginning of the 21st century, advances in telecommunications and the Internet, as well as increased access to computers and other digital devices, accelerated the uptake of digital learning. In health care, digital education is a well-accepted methodology of teaching in undergraduate studies, as well as a means to deliver continuous professional education to busy health care providers [1,2].

Digital education is “the act of teaching and learning by means of digital technologies” [3]. This is an overarching definition that encompasses a variety of educational approaches, concepts, methods, and technologies that are constantly evolving. Digital education can be classified according to the teaching methods, specifications of the technology, or modality of digital education. It can also be delivered as a stand-alone instructional method or as a blended-learning approach, combining elements of digital education with traditional, face-to-face learning [3]. Digital education comprises a wide range of interventions that can be broadly divided into the following [4-6]:

1. Offline and computer-based digital education (ie, offline digital education)
2. Online and local area network (LAN)-based digital education (ie, online digital education)
3. Digital game-based learning
4. Massive open online courses (MOOCs)
5. Psychomotor skills trainers, virtual reality environments, and virtual patient simulations
6. Mobile learning (m-learning)

Offline computer-based digital education refers to applications that do not require an Internet connection to deliver the learning activities, such as CD-ROMs, USB sticks, or material that was previously downloaded from a networked connection but does not require it for the learning activity [5]. Online digital education, also known as Web-based or LAN-based digital education, requires an active network connection to be delivered and it includes tutorials, discussions, or live conferencing, among other formats [7,8]. Digital game-based learning applies gaming principles and mechanics to create engaging learning activities to improve learners’ attitudes, motivation, and knowledge [5]. MOOCs are courses offered online to large numbers of participants, independently of their location and entry qualifications [9]. Psychomotor skills trainers, virtual reality environments, and virtual patient simulations are different approaches offering a first-person active learning experience. Psychomotor skills trainers are used to develop fine motor coordination skills and techniques, while virtual reality environments and virtual patient simulators are computer-generated depictions of a given environment or a patient clinical case, respectively [5]. Lastly, m-learning refers to any digital education intervention that utilizes mobile devices to deliver educational content [5].

Several characteristics of digital education are believed to improve knowledge, including flexibility to access study materials at a convenient place and time, interactive lessons that can be repeated according to the learners’ needs, and the availability of practice exercises with feedback to improve understanding [7,10-12]. Another characteristic that may be especially valuable for health care is the opportunity to provide lessons with standardized content that could help to produce globally accepted health care professionals [13]. These attributes make digital education particularly well-suited to deliver continuous medical education (CME) programs to professionals after entering the workforce. CME programs convey significant advantages to the skills and knowledge of health care providers, who generally understand their benefits and are willing to engage in these programs [14] despite facing significant barriers, such as lack of time due to work commitments, distance from educational centers, and costs [15,16].

Access to CME programs is particularly relevant to train professionals involved in the management of complex conditions, such us chronic wounds. Chronic wounds are wounds that “fail to proceed through an orderly and timely process to produce anatomic and functional integrity” [17]. They affect about 1% of the population, primarily the elderly, and are associated with a considerable reduction in the quality of life of affected patients [18]. Chronic wounds management requires intensive use of resources and personnel, particularly specialized nurses, increasing the economic burden on already overstretched health systems [19,20]. A key barrier to effective wound care appears to be a lack of interest in quality chronic wound care shown by health care professionals, a situation that can only be improved with revised, evidence-based wound care education [20]. A 2005 review by Flanagan et al on the barriers of implementation of evidence-based best practice in wound care highlighted the need for continuous education of nursing personnel, to overcome barriers to learning and develop critical skills [21].

Considering the immense economic and psychosocial burden of chronic wounds [19,22,23], we believe that a systematic evaluation of the effectiveness of digital education interventions to deliver learning programs on chronic wound management is required. A number of systematic reviews, including several already published or submitted by our group, have evaluated the effects of digital education interventions on different aspects of health professions’ education [4-6,24-36]. Nevertheless, to date, we have not encountered other literature or systematic reviews evaluating the effect of digital education interventions to train health care professionals on management of chronic wounds. Therefore, in this systematic review we will attempt to summarize the literature evaluating the use of digital education interventions to improve the management of chronic wounds by health care professionals.  

The objective of this systematic review is to assess the effectiveness of digital education for chronic wounds management as a stand-alone approach or as part of a blended-learning approach in pre- and postregistration health care professionals.

## Methods  

We will follow the Cochrane guidelines to conduct this systematic review [37]. A detailed summary of the methods we will use in this review were reported in a previous paper [3]. This protocol was registered with PROSPERO on October 9, 2018 (registration ID: CRD42018109971).

### Criteria to Select Studies to be Included in the Review

Our systematic review will include clinical trials in which any category of digital education intervention was utilized to train health care professionals in chronic wound management. The following study designs will be included: randomized controlled trials (RCTs), cluster RCTs, and quasi-RCTs. Cross-over trials will be excluded due to high risk of contamination as a result of carry-on effect. We will include eligible papers published in any language and in any type of publication, including research articles, abstracts, and conference proceedings.

The populations included in this review will include the following: (1) preregistration students pursuing a degree in any health care-related field, in a university or tertiary institution recognized by relevant governmental or professional bodies or (2) postregistration health care professionals, as referred by the Health and Welfare chapter of the International Standard Classification of Education: Fields of education and training 2013 (ISCED-F 2013) [38]. The health care-related professions eligible for the review include the following: medicine, nursing and midwifery, medical diagnostic and treatment technology, and therapy and rehabilitation. If a study presents data on more than one professional group or includes pre- and postregistration participants, it will be included if they report the results for each subgroup separately. Studies presenting a mixed-group analysis will be excluded. The included studies should present any of the comparisons that are listed in Textbox 1.

### Outcome Measures  

#### Primary Outcomes  

Aligned with previous systematic reviews on digital education interventions from our group, we will evaluate the following primary outcomes:

1. *Learners’ postintervention knowledge*, defined as the objective evaluation of learners' conceptual understanding, using validated or nonvalidated instruments, such as multiple-choice questionnaires or other kinds of questionnaires. If multiple posttest assessments were conducted, we will use the first posttest assessment in the analysis. Subsequent posttest assessments (ie, knowledge retention) will be used in sensitivity analysis (see Data Synthesis section below).
2. *Learners’ postintervention skills*, defined as the learners' ability to execute a procedure or technique (ie, management of chronic wounds, in this review) taught to them, assessed using any validated or nonvalidated instrument (eg, number of mistakes made, or time spent in the task).
3. *Learners' postintervention attitude*, defined as the learners' perceptions about the intervention and about patients and colleagues, in relation to acquiring new knowledge or skills. We will measure them using any validated or nonvalidated instrument as reported in the primary study.
4. *Learners' postintervention satisfaction*, defined as learners' levels of expectation and enjoyment toward the intervention, assessed using validated or nonvalidated instruments.
5. *Learners’ postintervention behavior change*, defined as any change in the way learners modify their practice or the way they interact with patients, measured using any validated or nonvalidated instrument.

**Comparisons that should be present in included studies.**

- Any digital education intervention versus traditional learning
- Any digital education intervention versus blended-learning intervention
- Any digital education intervention versus another digital education intervention
- Any digital education intervention versus no intervention
- Any blended-learning intervention versus traditional learning
- Any blended-learning intervention versus no intervention

#### Secondary Outcomes  

We will evaluate the following secondary outcomes:

1. *Patient-related outcomes*, as reported in the primary studies, will be assessed only in studies involving postregistration health care professionals using any validated or nonvalidated instruments.
2. *Cost and cost-effectiveness* of implementing the digital education interventions.
3. *Adverse effects of the digital education intervention*, including dropouts, isolation, and effects of isolation on the learners’ mental well-being (eg, depression and anxiety) and other adverse effects as reported in the primary studies.

### Identification of Studies  

This systematic review is one in a series of systematic reviews our group is conducting to assess the use of digital education modalities for pre- and postregistration health professionals’ education and training. Each review of the series addresses a different aspect of digital education, including categories (ie, online, offline, virtual reality, etc) [24,25,27,28,30,31], specific pathologies [26,29,32], or learning theories [33,34]. This systematic review will evaluate the use of digital education interventions for chronic wound management training. We will therefore utilize the same literature search as other reviews already completed or under development. The search strategy includes the databases listed in Textbox 2.

All databases were searched for studies published from January 1990 to August 2018 without any language restriction. We selected the year 1990 as the starting date of our search, since computer usage was limited to basic functions before that time. The search strategy was developed for MEDLINE and was later adapted to the other databases. Multimedia Appendix 1 presents the MEDLINE search strategy. We also searched the International Clinical Trials Registry Platform Search Portal and the Current Controlled Trials metaRegister of Controlled Trials for unpublished clinical trials to try to mitigate publication bias. Finally, we will examine the reference lists of all included studies and relevant systematic reviews, as well as perform a hand search of relevant journals. In the event that data retrieved from the published studies is incomplete or missing, we plan to contact the study authors to request clarification. The search results from all databases have been imported into a single EndNote X8.1 (Clarivate) library and duplicate records were removed.

Two authors will work in parallel to screen titles and abstracts to identify studies for full-text revision. The full-text versions of selected articles will be retrieved and assessed by two reviewers working independently. Reviewers’ individual results at each step of the screening process will be compared; disagreements will be settled between them or through an arbiter if an agreement cannot be reached. The steps of the screening process will be presented in a flow diagram according to the Preferred Reporting Items for Systematic Reviews and Meta-Analyses (PRISMA) statement [39], including the reasons for exclusion of papers at the full-text screening stage.

### Data Extraction and Quality Assessment

Two reviewers, working independently, will extract the data for all included studies using a Microsoft Excel prepiloted, standardized data recording form used by the group in other digital education systematic reviews. The information to be extracted includes the following: study design and participants’ demographics, type of digital education intervention, method and device used to deliver the intervention, and type of content (eg, images, text or video, and reported outcomes). Disagreements between the authors will be resolved through consensus or consultation with a third review author, considered the arbiter.

The methodological quality of included papers will be assessed in parallel by two authors using the *Risk of Bias* tool from Cochrane [40]. We will assess the following domains: random sequence generation; allocation sequence concealment; blinding of outcome assessment; completeness of outcome data; selective outcome reporting; and other sources of bias such as baseline imbalance, inappropriate administration of an intervention, or contamination. We will not assess blinding of participants or personnel, as the nature of the intervention precludes blinding. If we include cluster RCTs, the assessment will include the following: recruitment bias, baseline imbalance, loss of clusters, incorrect analysis, and comparability with individually randomized trials. Each parameter will be classified as high, low, or unclear risk of bias, using the words *yes*, *no*, or *unclear* and the colors red, green, and yellow, respectively. We will report the results using a risk-of-bias table or summary as per the Cochrane Handbook for Systematic Reviews of Interventions [41].

**Databases included in the search strategy.**

- MEDLINE (Ovid)
- Cochrane Central Register of Controlled Trials (CENTRAL; Cochrane Library)
- Embase (Ovid)
- Web of Science
- Educational Resource Information Centre (ERIC; EBSCO)
- PsycINFO (EBSCO)
- Cumulative Index of Nursing and Allied Health Literature (CINAHL; EBSCO)
- ProQuest Dissertation and Theses database

### Statistical Analysis

We will use Cochrane’s RevMan 5.3, the software used for preparing and maintaining Cochrane Reviews, to analyze the data. To estimate the effect size of the digital education interventions in the primary study, we will first calculate the mean difference and 95% CI if the results are reported as a continuous variable. We will calculate the risk ratio and 95% CI when the study reports the outcome as a dichotomous variable. If the same outcome is reported by more than one study using different measurement tools, we will recalculate mean differences into standardized mean differences. In the event that an RCT presents more than one intervention arm, the relevant digital education arm will be compared with the least-active control arm. If cluster RCTs are included in this review, we will aim to obtain data at the student level. If that is not possible, we will first establish if the original analysis had been adjusted for the effects of clustering and, if so, we will extract and use the reported estimates. Otherwise, we will check for unit of analysis errors and we will attempt to reanalyze the data using the appropriate unit of analysis and account for the intraclass correlation coefficients [42].

When a primary study is missing relevant outcome data, we will attempt to obtain the information by contacting the study authors. If a response is not obtained, we will report it accordingly. We will not impute any missing outcome data. We will, whenever possible, conduct analyses on an intention-to-treat basis.

### Assessment of Reporting Biases  

If the systematic review includes more than 10 studies, we will assess publication bias through a qualitative analysis of the characteristics of included studies using a funnel plot and regression weighted by the inverse of the pooled variance [43].

### Data Synthesis  

If the characteristics of the included studies allow, we will attempt to perform a meta-analysis. To proceed to the analysis, we will group the articles according to study design and type of intervention. We will categorize the studies’ outcomes as per Miller’s classification of clinical competence [44] to assess learners’ knowledge and skills in accordance to the type of assessment utilized (eg, if an outcome reported as *skill* is assessed by a knowledge test, we will consider the outcome as knowledge), independently of the teaching method. Leaners’ attitudes will be divided into cognitive, behavioral, or affective attitudes and analyzed independently [45]. Learners’ satisfaction will be reported in a narrative synthesis.

Before attempting the meta-analysis, we will assess if it is feasible by evaluating the included studies for methodological and statistical heterogeneity. We will assess the characteristics of the forest plot and calculate the *I*^*2*^ statistic [37]. If we observe substantial heterogeneity (ie, *I*^*2*^ greater than 0.5), we will explore its causes by conducting subgroup analysis. If extensive clinical or methodological heterogeneity is identified, we will not report a meta-analysis, but will instead use a narrative synthesis.

If a meta-analysis is possible, we will use a random-effects model, as it provides a more conservative estimate of effect and it is the preferred method when there is moderate heterogeneity. We will perform separate analyses for interventions among pre- and postregistration health care professionals. We will include the intention-to-treat analysis of the results in the meta-analysis.

To examine the impact of bias on study outcomes and in the results of the meta-analysis, we plan to perform sensitivity analyses. We will exclude studies according to the criteria in Textbox 3.

If the data allow, we plan to conduct subgroup analyses, stratifying the data as described in Textbox 4.

**Criteria to exclude studies for sensitivity analysis.**

- High risk-of-bias studies, as per our assessment using Cochrane’s Risk of Bias tool; we plan to meta-analyze the data, excluding high risk-of-bias studies, to examine the strength of the results
- Small studies with less than 30 participants in each study arm
- Source of funding as follows:

- Studies funded exclusively through industry sponsorship
- Studies funded through public and industry sponsorship that includes the free provision of study materials
- Studies not funded by industry sponsorship, including publicly funded studies and studies that did not provide free materials, or when the funding was not described or was unclear

- Studies comparing more than one digital education or blended-learning intervention to traditional learning; in this case, a sensitivity analysis will be performed to assess the impact of each intervention on the measure of effect

**Stratification of data for subgroup analyses.**

- Type of digital education intervention
- Chronic wound type: vascular ulcers (ie, venous ulcers and arterial insufficiency ulcers), diabetic foot ulcers, and pressure ulcers
- Registration stage: preregistration students and postregistration professionals
- Type of student or professional group, as per the International Standard Classification of Education: Fields of education and training 2013 (ISCED-F 2013) [38]
- Quartiles of adherence and time spent on the intervention, reported as a percentage
- Countries’ income—low- and middle-income countries versus high-income countries—according to the World Bank’s classification

### Reporting of Results

We will produce a narrative synthesis of results, even if a meta-analysis is not possible. The report will include a *Summary of Findings* table following the Cochrane Handbook for Systematic Reviews of Interventions [41]. The table will outline the main outcomes for each included study and, if a meta-analysis is possible, the table will present the results for each of the primary outcomes, as well as potential adverse effects, if they were reported by the primary studies.

## Results

We have completed the screening of titles and abstracts for this systematic review and are currently selecting papers against our inclusion and exclusion criteria through full-text revision. We are expecting to complete our review by the end of April 2019.

## Discussion

Digital technologies are increasingly used to deliver learning programs to pre- and postregistration health care professionals. Evidence from systematic reviews have shown that these programs are at least as effective as traditional learning in improving learners’ outcomes [46,47]. Digital learning offers a suitable alternative to deliver CME programs to health care professionals that may not be able to access them otherwise, due to work load, distance from learning centers, or costs [16]. This, in turn, may increase the uptake of these programs and potentially improve the quality of care. There is a wealth of evidence supporting continuous training to health care providers, particularly nursing personnel, in the management of chronic wounds [48-50]. As the global population ages, the burden of chronic wounds will continue to increase, making it crucial to ensure health care providers caring for these patients are properly trained and are following established, research-based practices.

Our systematic review will use stringent methodology to review the available literature and aim for an informed conclusion on the value of providing technology-enhanced education programs to enhance the quality of chronic wound management.

## Appendix

Multimedia Appendix 1 MEDLINE search strategy.

## Abbreviations

CENTRAL: Cochrane Central Register of Controlled Trials
CINAHL: Cumulative Index of Nursing and Allied Health Literature
CME: continuous medical education
ERIC: Educational Resource Information Centre
ISCED-F 2013: International Standard Classification of Education: Fields of education and training 2013
LAN: local area network
MOOC: massive open online course
PRISMA: Preferred Reporting Items for Systematic Reviews and Meta-Analyses
RCT: randomized controlled trial
